# Leukemic retinophaty, the first manifestation in a case of acute myelogenous leukemia

**Published:** 2018

**Authors:** Oana Roxana Scripcă, Cristina Pădurariu, Neacșu Gheorghe Boricean, Loredana Botoș

**Affiliations:** * Ophthalmology Department, County Emergency Hospital, Brașov, Romania; ** Haematology Department, County Emergency Hospital, Braşov, Romania

**Keywords:** LAM3-Acute promyelocytic leukemia, bone marrow aspiration and biopsy, leukemic retinopathy, sepsis, disseminated intravascular coagulation (DIC), all-trans retinoic acid

## Abstract

A 42-year-old woman, without a specific medical history presented at the Department of Emergency Ophthalmology accusing marked decrease of vision for the left eye (VA 1/ 100). The eye examination revealed an optic neuropathy with multiple retinal hemorrhages at the level of both eyes, but more acutely on the left eye. The brain computer tomography (CT) excluded the suspicion of increasing intracranial pressure. The common blood tests such as complete blood count (CBC), erythrocyte sedimentation rate, and inflammatory markers raised a high suspicion of a malignant haematological disease.

## Introduction

Two of the most common pathologies investigated in ophthalmology are optic disc oedema and retinal vascular diseases. In this context, the imagistic evaluation often transcends the diagnosis, the clinician having high-performance technology in his hands, such as CT, MRI, OCT, but there are situations in which common assays such as CBC and the inflammatory markers can be the only medical investigation that clearly suggests the existence of a life-threatening haematological disorder.

## Case report

We present the case of a 42-year-old female patient with no significant pathological history. She presented at the Department of Emergency Ophthalmology, claiming a drop of vision in the left eye (LE), which gradually installed during the last 48 hours before she presented at the Emergency Department. There was no history of ocular trauma or surgery.

The ophthalmic evaluation at the time of admission revealed a well-contoured and normally colored optic disc, retinal hemorrhage radiating from the inferotemporal optic disc margin with a flame shaped aspect, dot-spot retinal hemorrhages and low foveolar reflex, at the right eye (RE). At the left eye (LE), an elevated optic disc with moderate retinal oedema marked retinal hemorrhages, and preretinal hemorrhage with no foveolar reflex. The slit lamp examination of the anterior segment was normal for the RE and delayed RFM for the LE.

The visual acuity (VA) for the RE was 0.9 with correction and for the LE was counting fingers at 1 meter, not correctable.

In the first stage of biohumoral status assessment, we performed blood tests such as CBC, erythrocyte sedimentation rate, C-reactive protein, fibrinogen, blood glucose test, lipid profile, total serum protein and thyroid markers. The results of blood test showed a severe pancytopenia with 1.43 X 10³/ µl leukocytes, 24 X 10³/ µl thrombocytes and severe low levels of Hb 5 g/ dl, inflammatory markers were high, erythrocyte sedimentation rate ESR 60 mm/ h, CPR 17.63 mg/ l, lower fibrinogen levels 94.77 mg/ dl. Given the analyses, we suspected disseminated intravascular coagulation and we performed D- Dimeri test with positive result.

Interdisciplinary medical evaluations were made by colleagues from internal medicine, cardiology and hematology departments. In the multidisciplinary team, we decided to complete the investigations with Hemostasis Analyzer Markers (Pts, Ptr, INR, and APTT), which suggested a hemostasis disorder. Lactate dehydrogenase test, Polymerase chain reaction (PCR) and ferritin were on high levels, serum iron was on low levels, negative serology for HIV, Hepatitis B and Hepatitis C, anti-thyroid peroxidase antibodies (anti-TPO antibodies) and the thyroid hormones within normal limits. At the time of admission, the CT for head segment did not indicate pathological changes.

**Fig. 1:**
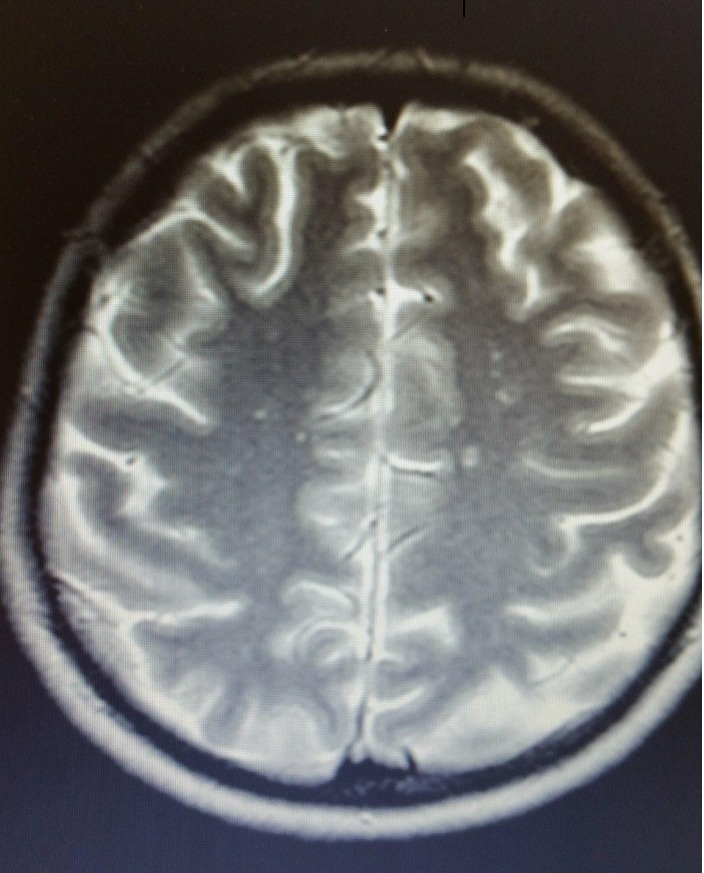
CT for head segment

A bone marrow aspiration and biopsy were made. Leukocyte concentrate, cytological blood smear test, and the cytological examination of the spinal cord were inconclusive because they showed a low percentage of promyelocytes of approximately 8% and blasts of approximately 6%. Due to the patient’s general condition changing with the onset of fever we decided to transfer her to the intensive care unit. Given the unfavorable evolution, we repeated the CT for head segment, which showed multiple supratentorial lesional processes predominantly in the white matter and calf body and a few isolated thalamic lesions that raised the suspicion of brain microabscesses in a septic context (**Fig. 1**). The head CT also revealed a modulation of signal diffusion in the left eye-viewable on the hemosiderin-susceptible sequence in moderate hyposignal, which almost totally interests the vitreous body, possibly hemorrhagic.

At the CT for thorax and abdomen, we found multiple pulmonary outbreaks spread in both lungs, parapneumonic pleurisy of 6 mm on the right and 5 mm on the left, pelvic ascites fluid with infiltrative appearance around the genitals. Blood culture test was negative; RT-PCR Sepsis test was negative for S. aureus, Enterococcus faecalis, Streptococcus agalactiae, Enterobacteriaceae, Candida spp and Pseudomonas aeruginosa. 

**Fig. 2:**
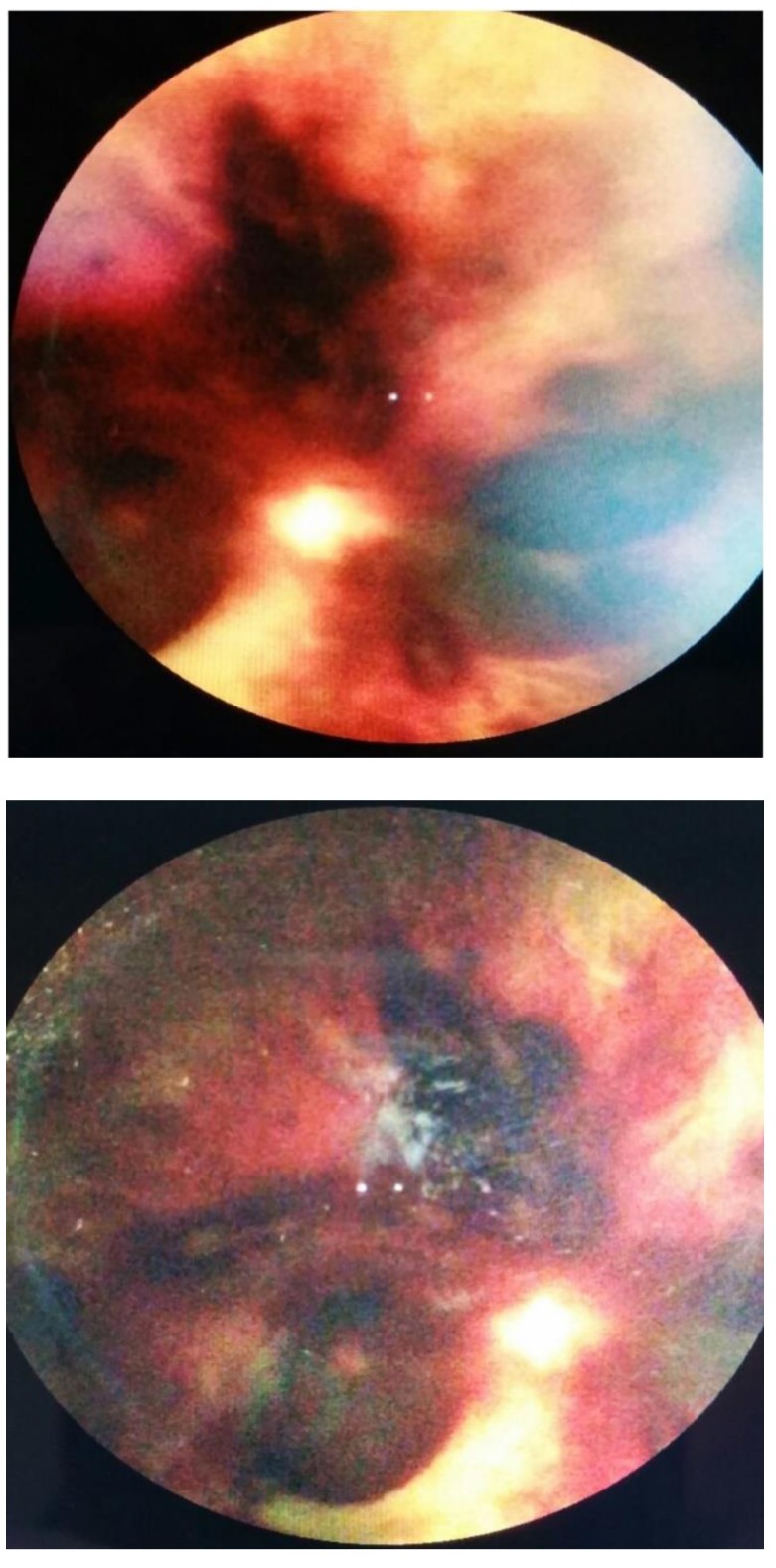
Fundus of the right eye, showing a massive retinal hemorrhage 6 days after admission

**Fig. 3:**
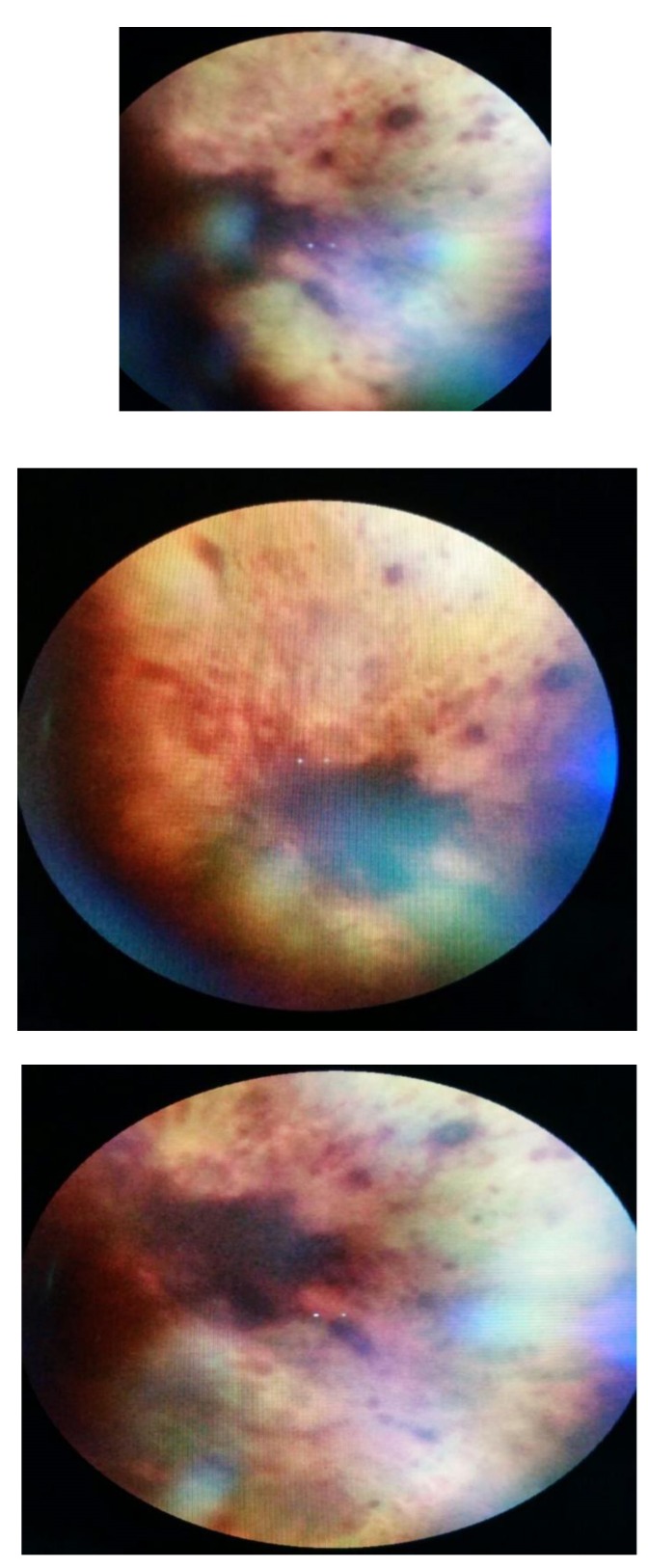
Fundus of the left eye, showing a big retinal hemorrhage that covers the optic disc and multiple dot-spot hemorrhages, 6 days after admission

The examination of the posterior pole revealed marked changes of the retina and optic disc with massive hemorrhages that practically covered a large part of the posterior pole for the LE and for the RE extensive retinal hemorrhage, marked papillary edema, both eyes with vitreous condensation (**Fig. 2**,**3**). It was difficult to determine the patient’s VA given the general altered status.

**Fig. 4:**
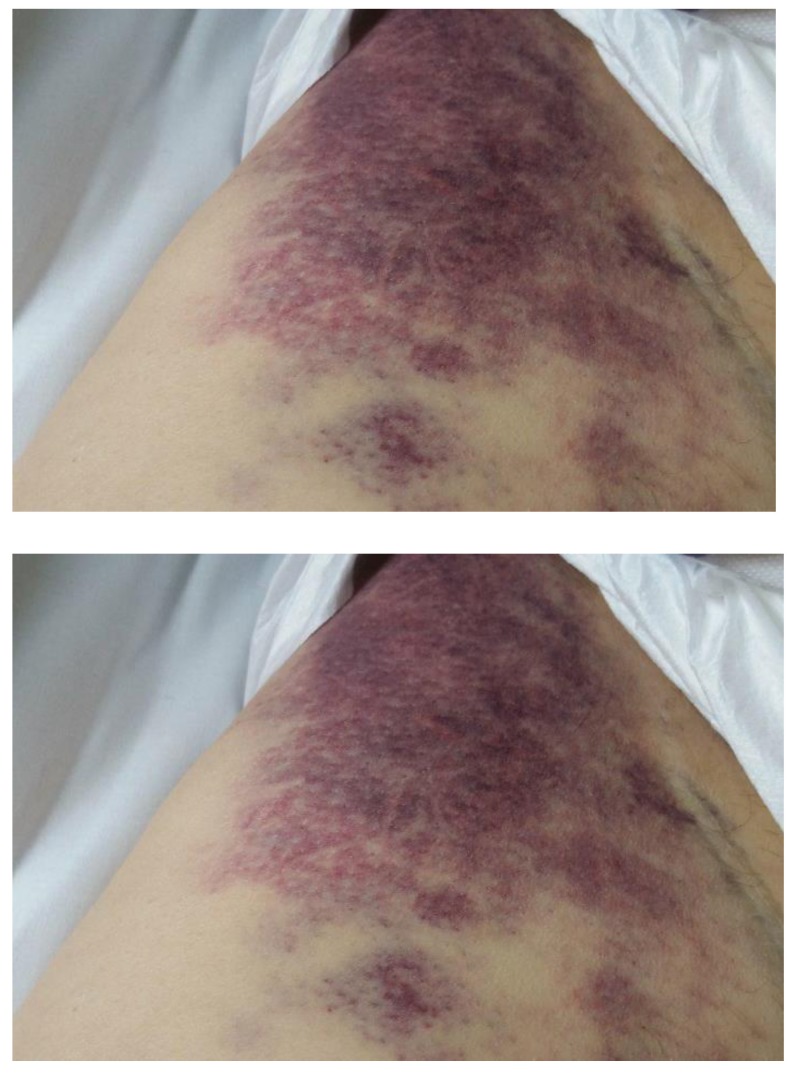
Extended cutaneous hemorrhage syndrome

The patient was transferred to the hematology department having an extended mucosal cutaneous hemorrhage syndrome (**Fig. 4**), hepatic cytolysis syndrome and started the specific treatment for LAM3 with Vesanoid 45 mg/ m2 - 80 mg/ day. The general status of the patient worsened, becoming confused, jaundice with haematological status in the collapse with WBC=55.76 X 10³/ µl, Neutrophils 0.00 X 10³/ µl, Lymphocytes 0.00 X 10³/ µl, Monocytes 0.00 X 10³/ µl, Eosinophils 0.00 X 10³/ µl, Basophils 0.02 X 10³/ µl, she had PLT=8 X 10³/ µl, Hb=7.7 g/ dl.

In the hematology department, the test battery was extended with flow cytometer, PML RAR alpha transcript t 15, 17, and immunohistochemistry. The result of PML RAR alpha screening transcript (15;17) was positive.

The patient received antibiotics, red blood cell transfusion, freshly frozen plasma platelets, cryoprecipitate, corticosteroid, and haemostatic. Due to severe coagulopathy and severe fibrinogenemia, she received treatment with Haemocomplettan fibrinogen concentrate.

Thirteen days after admission, the patient suffered a respiratory cardiac arrest and died. Later on, after the patient died, the result of Bone Marrow Biopsy revealed a hypercellularity with intertrabecular infiltration of blastic cells with cytoplasmic granulations and Auer rods, about 80-90% of the total cells.

## Discussions

***Background***

Acute promyelocytic leukemia is the type of acute leukemia with myeloid population dominated by the promyelocytes. There are two types of classification, the French-American-British (FAB) composed of the morphological and cytochemical aspects of the cells notes the acute promyelocytic leukemia as AML M3 and the World Health Organization (WHO) classification system noted as APL with translocation between chromosomes 15 and 17, t(15;17) [**1**,**2**].

The first reference about APL associated with disseminated intravascular coagulation (DIC) was made in 1959 by Jean Bernard, who described the disease like a hyperacute fatal illness associated with a hemorrhagic syndrome [**3**].

**Fig. 5:**
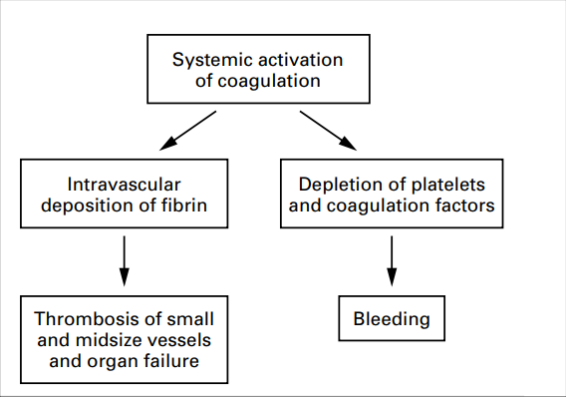
The Mechanism of disseminated intravascular coagulation [**4**]

Disseminated intravascular coagulation is a haematological syndrome characterized by the activation of circulating thrombin and plasmin, which can cause thrombosis and hemorrhage in the small and midsize vessels (**Fig. 5**). Depending on the haematological and metabolic status of the patient with disseminated intravascular coagulation, the failure of multiple organs may occur [**4**].

The clinical pathology associated with disseminated intravascular coagulation is myeloproliferative diseases, trauma, and sepsis.

***Course***

The patients with LAM3 diagnostic are generally young. The onset is sudden with hemorrhagic syndrome. It is characterized by mucosal, cutaneous hemorrhage and thrombotic manifestations.

DIC and thrombosis can be a clinical manifestation in LAM3. Clinically deep thrombophlebitis, pulmonary embolism, cerebral thrombosis, and renal thrombosis occur. In this situation, secondary fibrinolysis is damaged or there is a vascular lesion, which favors accumulation of fibrin. Hypercoagulability is induced by tissular factors of the leukemic M3 cells. This factors binds of FVII in the presence of Calcium and activates the X factor (extrinsic pathway) inducing thrombin and then fibrin production [**5**]. 

LAM3 is a medical emergency; therefore, the treatment should be initiated as soon as possible. In the absence of treatment, the prognosis is very poor [**1**].

The leukocyte count prior to the onset of therapy is the most relevant prognostic factor for survival (high risk WBC > 10.000/ µl). Age is another important factor. Prognosis of LAM3 after treatment is favorable with very high rates of complete remission, more than 90% [**6**].

***Differential diagnosis***

Differential diagnosis is mainly made with other type of acute leukemia especially LAM2 and LAM4, aplastic anemia, folic acid deficiency and myelodysplastic syndrome. 

Pancytopenia can be caused by a large variety of diseases of varying severity, including vitamin deficiencies, agranulocytosis with arrested maturation at the promyelocyte stage and autoimmune disease. It is important to review the peripheral blood smear at the time of initial evaluation of all patients with hematologic disorders [**7**].

Diagnosis relies on laboratory findings showing pancytopenia or hyperleukocytosis in around 20% (complete blood cell count with differential cell counts), bone marrow examination that shows large blasts, numerous granules, Auer rods often in bundles, faggot cells, promyelocytic cells, and strong positive on myeloperoxidase staining, bone marrow histology and on coagulation status [**7**].

Immunophenotyping of promyelocytic cells shows positivity for CD13, CD33, CD117, MPO, and negativity for HLA-DR, CD 2, and CD 34 [**7**].

The diagnosis is confirmed by molecular screening (RT-PCR), immunofluorescence anti-PML antibody and cytogenetic or FISH (fluorescence in situ hybridization) analysis of PML/ RARA fusion genes [**7**].

***Prognosis***

A study made by Reddy, Quah, Low and Jackson [**8**] on seventy-seven patients with acute leukemia who presented intraretinal hemorrhages (IRH), white-centered hemorrhages, cotton-wool spots and macular hemorrhages, concluded that the hemorrhagic pathological changes in the retina lead to a poor prognosis [**8**]. 

***Treatment***

Treatment of acute promyelocyte leukemia should consider both the increased frequency of DIC, which requires sustained therapy as well as the therapeutic response to a non-cytostatic agent: isotretinoin (all-trans retinoic acid, ATRA). ATRA 45 mg/ m2/ day P.O. can induce full remission within the induction protocol in patients with LAM3 possessing translocation t(15;17). Remission is not long lasting and therefore LAM classical chemotherapy should be associated to the treatment [**9**].

## Conclusions

There are a few pathologies described in literature that associate bilateral retinal hemorrhagic changes. C Ling, PL Atkinson, and CG Munton [**10**] mention the appearance of bilateral retinal hemorrhages following epidural injection. A case of acute bilateral retinal hemorrhages occurring after injection of oxygen-ozone (O2O3) mixture for lumbar disc herniation was described by Giuseppe Lo Giudice, Franco Valdi, Maurizio Gismondi, Giovanni Prosdocimo and Valentinade Belvis [**11**] in American Journal of Ophthalmology, 2014.

There are situations in which a subarachnoid hemorrhage (SAH) or traumatic brain injury can cause retinal changes either by direct blood flow to the optic nerve sheath or by cerebrospinal fluid (CSF) leakage in the optic nerve sheath, which will cause compression in the central vein of the retina and subsequently retinal hemorrhage in the vitreous, subhyaloid or intraretinal/ sub-internal limiting membrane [**12**].

From a hematological point of view, the particularity of this case is that the diagnosis was based on bone marrow biopsy since the cytological examination of blood smear and bone marrow aspiration were inconclusive. After treatment with All-Trans Retinoic Acid (ATRA Vesanoid) and fibrinogen concentrate (Haemocomplettan), the disseminated intravascular coagulation did not resolve and the coagulopathy continued leading to the death of the patient.

Another particularity of the case is that although the patient presented at the hospital invoking only decreased vision, a thorough posterior pole examination revealed the presence of acute bilateral retinal vascular signs, these being the only hint that we might be dealing with in a hematological malignant disease. All these draw attention to the importance of routine blood tests that were the first steps for a prompt and correct diagnosis.
